# Human endogenous retrovirus-FRD envelope protein (syncytin 2) expression in normal and trisomy 21-affected placenta

**DOI:** 10.1186/1742-4690-5-6

**Published:** 2008-01-23

**Authors:** André Malassiné, Jean-Louis Frendo, Sandra Blaise, Karen Handschuh, Pascale Gerbaud, Vassilis Tsatsaris, Thierry Heidmann, Danièle Evain-Brion

**Affiliations:** 1 INSERM, U767, 4 avenue de l'Observatoire 75006 Paris, France; 2 Université Paris Descartes, Faculté des Sciences Pharmaceutiques et Biologiques, 4 avenue de l'Observatoire, 75006 Paris, France; 3 CNRS, Paris, F-75006 France; 4 Unité des Rétrovirus Endogènes et Eléments Rétroïdes des Eucaryotes Supérieurs UMR 8122 CNRS, Institut Gustave Roussy, 39 rue Camille Desmoulins, 94805 Villejuif, France; 5 PremUP, Paris, France

## Abstract

Human trophoblast expresses two fusogenic retroviral envelope proteins, the widely studied syncytin 1, encoded by HERV-W and the recently characterized syncytin 2 encoded by HERV-FRD. Here we studied syncytin 2 in normal and Trisomy 21-affected placenta associated with abnormal trophoblast differentiation. Syncytin 2 immunolocalization was restricted throughout normal pregnancy to some villous cytotrophoblastic cells (CT). During the second trimester of pregnancy, syncytin 2 was immunolocalized in some cuboidal CT in T21 placentas, whereas in normal placentas it was observed in flat CT, extending into their cytoplasmic processes. *In vitro*, CT isolated from normal placenta fuse and differentiate into syncytiotrophoblast. At the same time, syncytin 2 transcript levels decreased significantly with syncytiotrophoblast formation. In contrast, CT isolated from T21-affected placentas fused and differentiated poorly and no variation in syncytin 2 transcript levels was observed. Syncytin 2 expression illustrates the abnormal trophoblast differentiation observed in placenta of fetal T21-affected pregnancies.

## Background

Human endogenous retroviruses (HERV) comprise approximately 8% of the human genome [1,2]. Most of the identified elements are defective due to mutations and/or deletions within their genes, but some elements have conserved intact open reading frames. A systematic search for non-defective endogenous retrovirus envelope protein genes has led to the identification of 16 genes [3]. Among them, two can induce cell-cell fusion when expressed in different cells and are highly and specifically expressed in the human placenta [4-6]. The products of these two genes are glycoproteins named HERV-W Env glycoprotein (syncytin 1) and HERV-FRD Env glycoprotein (syncytin 2).

**Figure 1:**
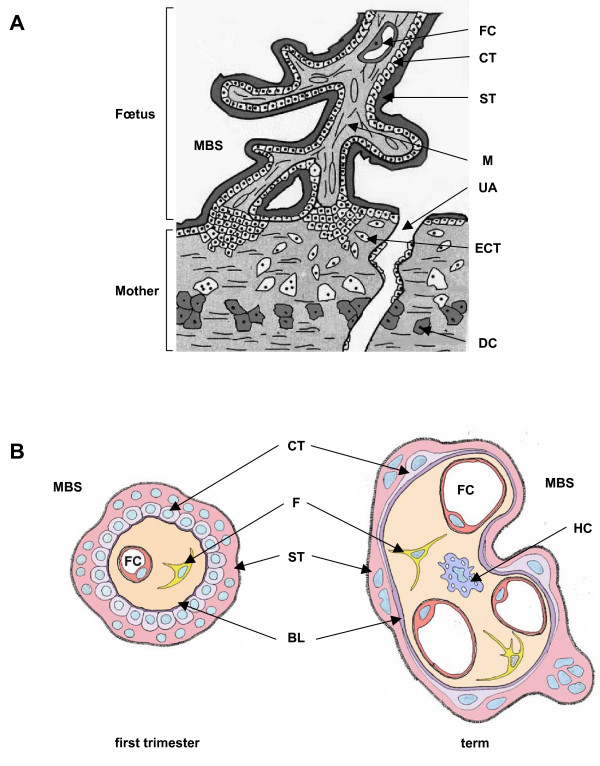
**A. **In humans, at 10–12 weeks of pregnancy, the chorionic floating villi are in contact with the maternal blood in the maternal blood space (MBS). In these villi, cytotrophoblastic cells (CT) differentiate by fusion to generate the syncytiotrophoblast (ST). In the anchoring villi the cytotrophoblastic cells proliferate and invade the decidua (DC). The extravillous cytotrophoblastic cells (ECT) invade the lumen of uterine arteries (UA). FC: Fetal capillary; M: mesenchyme. **B. **Evolution of human floating chorionic villi. The chorionic villi, in direct contact with the maternal blood in the maternal blood space (MBS), consist of cytotrophoblastic cells (CT) and syncytiotrophoblast (ST) surrounding a core of mesenchymal cells including fetal capillaries (FC), fibroblasts (F) and Hofbauer cells (HC). BL: basal lamina.

In the human placenta, the trophoblast differentiates along two major pathways both critical for normal placental function [7]. In the extravillous trophoblast invasive pathway, the cytotrophoblastic cells of the anchoring villi in contact with the uterus wall proliferate, detach from the basement membrane and aggregate into multilayered columns of non-polarized cells that invade the uterus wall (Fig. 1). These cells, which compose the extravillous cytotrophoblast (ECT), invade the endometrium, the first third of the myometrium and the associated spiral arterioles. In the villous trophoblast pathway, the cytotrophoblastic cells of the floating villi proliferate, differentiate and fuse to form a syncytiotrophoblast (ST) that covers the entire surface of the villi (Fig. 1). The syncytiotrophoblast layer plays a major role throughout pregnancy, since it is the site of numerous placental functions, including ion and nutrient exchange and the synthesis of steroid and peptide hormones required for fetal growth and development. This multinucleated syncytiotrophoblast is regenerated along pregnancy by a continuous turnover process including proliferation of underlaying mononuclear cytotrophoblasts (CT), fusion of these cytotrophoblasts into syncytiotrophoblast and progression toward apoptosis.

*In vitro *isolated mononuclear cytotrophoblasts aggregate and fuse together to form a non proliferative, multinucleated syncytiotrophoblast which secretes human chorionic gonadotropin (hCG) and human placental lactogen (hPL), specific to pregnancy [8,9].

Trisomy of chromosome 21 (T21), which causes the phenotype known as Down syndrome, is the major known genetic cause of mental retardation and is found in around 1:800 live births. Little is known about placental development in this aneuploid condition. However, a defect in syncytiotrophoblast formation in T21-affected placentas is observed. Cultured cytotrophoblasts, isolated from T21-affected placentas, aggregate but fuse poorly or belatedly [10-13].

Trophoblast fusion and differentiation directly involves different molecular actors [14]. Among them, the HERV-W envelope glycoprotein named syncytin 1 is expressed in all trophoblastic cells [15,16] and directly involved in human trophoblast fusion and differentiation [17]. HERV-FRD Env glycoprotein (or syncytin 2) has been more recently shown to be expressed in the human placenta [1,4,18]. The aim of this work was therefore to study *in situ *and *in vitro *the expression and localization of syncytin 2 in human placenta at different stages of gestation, in normal and T21-affected pregnancies.

## Results

### Syncytin 2 is immunolocalized in cytotrophoblastic cells throughout normal pregnancy

**Figure 2:**
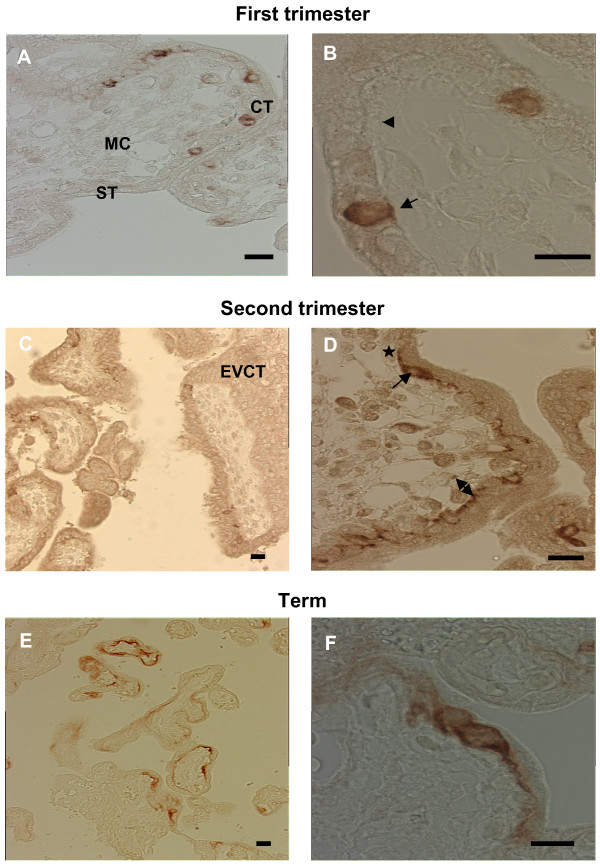
**Immunohistochemical analysis of syncytin 2 (HERV-FRD Env) in human placenta**. Top panel. First trimester floating villi (8 weeks of pregnancy). **A. **Syncytin 2 was detected in some cytotrophoblastic cells (CT). No immunostaining was observed in the syncytiotrophoblast (ST) and in the mesenchymal core (MC). Scale bar = 10 μm. **B. **This large magnification allows to clearly establish the cytoplasmic localization of syncytin 2 immunostaining in a pair of cytotrophoblastic cells. At this gestational age the cytotrophoblast consists of a continuous single layer of cuboidal cells beneath the syncytiotrophoblast. Scale bar = 10 μm. Arrowhead: non labeled CT, arrow: positively stained CT. Middle panel. Second trimester placenta (16 weeks of pregnancy). **C. **Immunostaining with anti-syncytin2 antibody shows positive reactivity in some cytotrophoblastic cells. No syncytin 2 reactivity was detected in the extravillous trophoblast (ECT), in the syncytiotrophoblast and in the mesenchymal core. Scale bar = 10 μm. **D. **In this floating villi, syncytin 2 immunostaining was observed in the cytoplasm of some cytotrophoblastic cells (arrow), in their thin cytoplasmic processes (star) and at the level of the trophoblastic basal lamina (double head arrow). Scale bar = 10 μm. Bottom panel. Term placenta floating villi. **E. **Syncytin 2 was detected in the cytoplasm surrounding the nuclei of flat cytotrophoblastic cells and in their thin elongated cytoplasmic processes. Staining was absent from some villi. Scale bar = 10 μm. **F. **This large magnification allows to clearly establish the syncytin 2 immunostaining continuity within cytotrophoblasts between the cytoplasm surrounding the nuclei and that of the thin cytoplasmic processes. Scale bar = 10 μm.

Figure 2 shows the syncytin 2 immunolocalization in chorionic villi throughout normal pregnancy. In first trimester placenta (Fig. 2A–B), syncytin 2 was only detected at the level of the cytotrophoblastic cells, which form a continuous single layer of cuboidal cells beneath the syncytiotrophoblast. Immunostaining was observed only in the cytoplasm of some cytotrophoblastic cells and never observed in the syncytiotrophoblast and in the mesenchymal core of the villi (Fig. 2A–B). In second trimester placenta (Fig. 2C–D), syncytin 2 immunostaining was present: 1/in the cytoplasm of cytotrophoblastic cells; 2/in their thin elongated cytoplasmic processes coming into contact with the syncytiotrophoblast and covering the villus basal lamina. At term (Fig. 2E–F), immunostaining was detected at low magnification in a fraction of the flat cytotrophoblastic cells and extended into their thin cytoplasmic processes. Higher magnification showed the continuity of syncytin 2 immunostaining between the cytoplasm surrounding the nuclei and that of the thin elongated cytoplasmic processes.

### Syncytin 2 immunostaining of cytotrophoblastic cells differs between T21 and gestational age-matched control placentas

**Figure 3:**
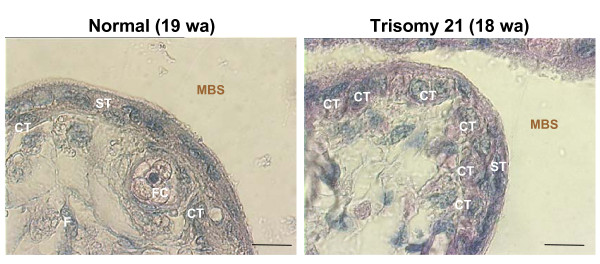
Second trimester chorionic villi of normal (19 weeks of amenorrhea: wa) and trisomy 21 (18 wa) placentae. In normal placenta, a large amount of cytotrophoblastic cells (CT) have fused into a thin multinucleated syncytiotrophoblast (ST). In trisomy 21 placenta, many cuboidal cytotrophoblastic cells (CT) are still present beneath the syncytiotrophoblast (ST) increasing the thickness of the trophoblastic layer. Scale bar = 10 μm.

**Figure 4:**
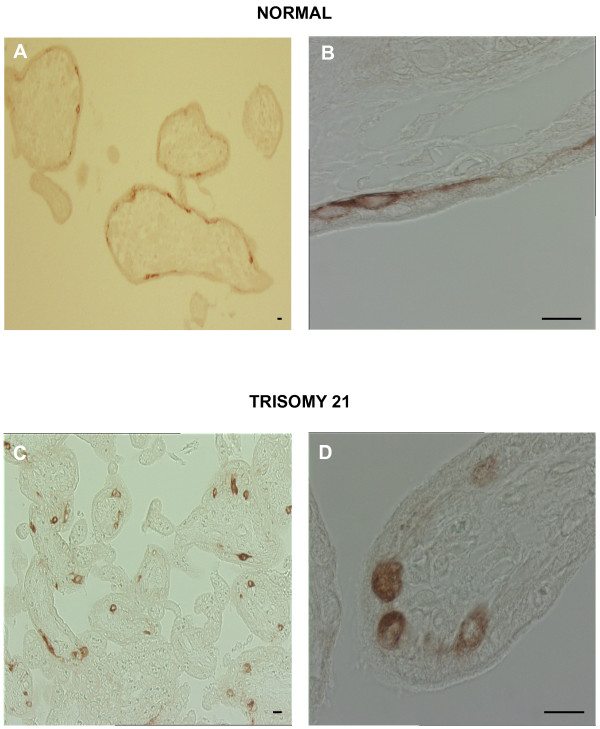
Immunohistochemical analysis of syncytin 2 (HERV-FRD Env) in age-matched second trimester (19 weeks) normal (upper panel) and T21-affected placentas (lower panel). Upper panel **A. **Immunostaining with anti-syncytin2 antibody showed positive reactivity in a fraction of elongated cytotrophoblastic cells. Scale bar = 10 μm. **B. **In this large magnification, syncytin 2 immunostaining was observed in the cytoplasm of cytotrophoblastic cells and in the thin cytoplasmic processes. Scale bar = 10 μm. Lower panel **C. **Syncytin 2 was detected in some cuboidal cytotrophoblastic cells. Scale bar = 10 μm. **D. **This high magnification shows the cytoplasmic localization of syncytin 2 immunostaining in several cytotrophoblastic cells. Scale bar = 10 μm.

A striking difference was observed between T21 second trimester placentas (18–19 weeks of gestation) and age-matched controls (Fig. 3). Indeed, as previously described, an increase in the thickness of the trophoblastic layer was observed in T21-placenta. As shown in figure 4, syncytin 2 was immunolocalized in some cuboidal cytotrophoblastic cells beneath the syncytiotrophoblast in T21 placentas (Fig. 4C–D). In gestational age-matched controls, syncytin 2 was observed in some flat cytotrophoblastic cells and extended into their thin elongated cytoplasmic processes (Fig. 4A–B), as already noted above.

### With time in culture Syncytin 2 transcript levels decrease in normal but not in T21 cells

**Figure 5:**
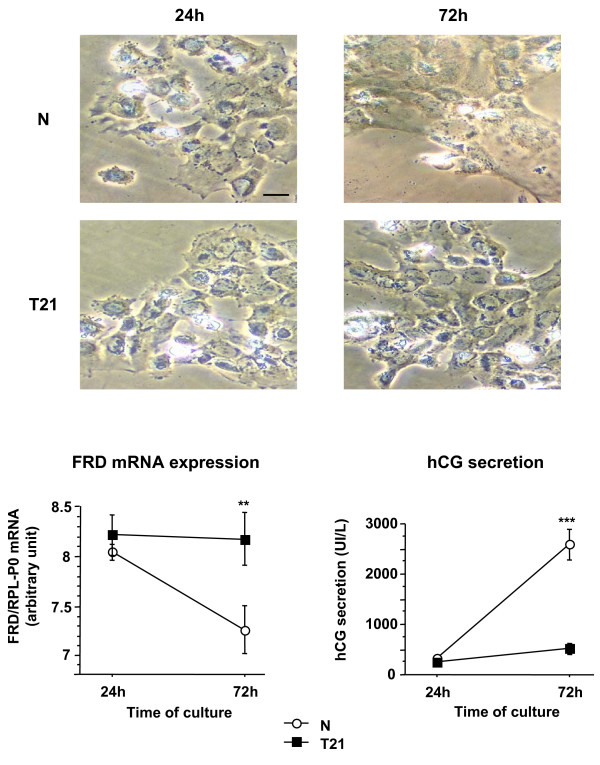
Morphological differentiation (upper panel), real-time RT-PCR analysis of *syncytin 2 *(HERV-FRD *env*) transcripts (lower left panel) and hCG secretion (lower rigth panel) during *in vitro *culture of control and T21 trophoblastic cells. Cytotrophoblastic cells were purified from three distinct age matched (second trimester) control and T21-affected placentas and separately cultured. The cells were visualized under phase contrast light microscopy (Scale bar = 10 μm). At 72 h, normal cytotrophoblastic cells had fused resulting in the formation of a large syncytium containing numerous nuclei. In contrast, T21 cytotrophoblasts were still aggregated and had not fused. Total mRNA were extracted after 24 and 72 h of culture. Data are expressed as the level of *syncytin 2 *mRNA normalized to that of *RPLP0 *mRNA. HCG secretion into the culture medium was measured at the indicated times, in normal (N) and T21-affected cell cultures. Results are the means ± SEM of triplicate dishes from three different cultures.

Cytotrophoblastic cells isolated from the chorionic villi of second trimester normal placentas adhere to plastic dishes, aggregate and fuse together to form a syncytiotrophoblast within 48 to 72 hours (Fig. 5 upper panel). Syncytin 2 positive immunostaining was only observed in some cytotrophoblastic cells forming aggregates; the staining was more intense at the sites of contact between these syncytin 2-positive, aggregated cells (data not shown). No immunostaining was observed in multinucleated syncytiotrophoblast after 48 or 72 hours of culture. Cytotrophoblastic cells isolated from T21-affected placentas adhered to the plastic dishes, aggregated, but did not fuse or fused only after a delay and poorly differentiated into syncytiotrophoblast (Fig. 5 T21). This was not associated with any significant increase in hCG secretion during the 72 hours of culture (Fig. 5 bottom panel). In these T21 trophoblastic cells, syncytin 2 transcript levels did not vary during the 72 hours of culture (Fig. 5 bottom panel). In contrast, in trophoblastic cells isolated from age-matched control placentas, the *in vitro *formation of syncytiotrophoblast was associated with a drastic increase in hCG secretion and syncytin 2 transcript levels decreased significantly (p < 0.02) between 24 and 72 hours of culture (Fig. 5).

## Discussion

The role of endogenous retroviruses in placental morphogenesis and trophoblast differentiation was hypothesized 10 years ago [19]. More recent studies point to the presence of HERV-R (ERV 3), HERV-FRD, HERV-W, HERV-F, HERV-K and HERV-T in human placenta, coding for intact retroviral Env proteins [3]. However the role of theses retroviral envelope proteins is still poorly understood.

A role for the ERV-3 envelope protein (produced by the single-copy human endogenous retrovirus ERV-3) have been suggested in trophoblast proliferation and differentiation [20,21]. However, the physiological knockout of the ERV-3 envelope (lacking both the fusion peptide and the immunosuppressive domain) in 1% of the Caucasian population [22] suggests that no essential function in placentation is associated with the expression of the ERV-3 envelope protein.

HERV-W envelope protein, syncytin 1, is directly involved in villous trophoblast fusion and differentiation [17]. The syncytin 1 is expressed in all trophoblastic cells, villous and extravillous trophoblast, independently of their differentiation stage [15,16].

In this study, we show that throughout pregnancy, HERV-FRD envelope protein, syncytin 2 is detected in some villous cytotrophoblastic cell and therefore this localization differs from syncytin 1 localization. All along pregnancy, the syncytiotrophoblast regenerates from the fusion of the underlying cytotrophoblasts. This process includes the continuous trophoblast turnover including proliferation of cytotrophoblast progenitors, the withdrawal of cytotrophoblasts from the cell cycle to G_0_, the recruitment of these post-mitotic cells to syncytiotrophoblast after membrane fusion and progression of syncytiotrophoblast towards apoptosis. Therefore the expression of syncytin 2 in some cytotrophoblastic cells suggest that it is expressed when the cytotrophoblastic cell is engaged in the fusion stage. As illustrated in second trimester placenta cytotrophoblastic cells are immunostained for syncytin 2 more frequently at the level of the cell membrane and this staining occurs at the sites of contact with the syncytiotrophoblast. Localization at this interface is precisely that expected for a protein directly involved in the fusion of the mononuclear cytotrophoblastic cells into the syncytiotrophoblast.

In addition, the syncytin 2 immunolabeling reflects the structural changes of the cytotrophoblastic layer during pregnancy. Indeed, as recently demonstrated, the cytotrophoblastic cell layer becomes thinner: the cuboidal cells are transformed to flat cells with many cellular processes that together with those of the adjacent syncytiotrophoblast eventually cover the basal lamina in a complex network of interdigitations [23].

In T21-affected placentas, localization of the labeled syncytin 2 differs notably from that in gestational age-matched control placentas. Syncytin 2 is mainly located in the cytoplasm of cuboidal cytotrophoblastic cells. This observation highlights *in situ *the delay in the fusion process of T21 trophoblastic cells and the delay in the maturation of the chorionic villi from T21-affected placentas [24,25] (Fig. 3).

In addition the transcript levels of syncytin 2 decreased significantly during *in vitro *differentiation of normal cytotrophoblastic cells into syncytiotrophoblast. In contrast in isolated T21 cytotrophoblastic cells, which did not fuse transcript levels of syncytin 2 did not vary with time in culture. Interestingly, we recently demonstrated that the *in vitro *defect of syncytiotrophoblast formation in T21 is reversible when cytotrophoblastic cells are treated with biosynthetic human chorionic gonadotropin. These results point to a major role of abnormal hCG and its receptor in T21 placental defect [26].

Envelope glycoprotein of HERV-W (syncytin 1) [17] interacts with its identified receptor RDR, also known as the neutral aminoacid transporter SLC1A5/ASCT2 [5,27]. Syncytin 2 entered the primate genome earlier than syncytin 1, namely before the split between New World and Old World Monkeys (i.e >40 Myrs ago). It also differs in its receptor, as demonstrated by *ex vivo *cell-cell fusion assays using different cell types [4]. The identification of this receptor and the direct role of syncytin 2 of in human syncytiotrophoblast formation need to be investigated.

Recently, other retroviral envelope proteins have been identified in placenta from other species. In mouse placenta two related *env *genes (syncytin A and syncytin B) were characterized [28] and it was demonstrated that the endogenous Jaagsiekte sheep retrovirus envelope regulates trophectoderm growth and differentiation in perimplantation ovine conceptus [29]. The role of these retroviral envelope proteins in fetoplacental development is still poorly known but their pleiotropic functions, including immunosuppressive activity argue for a critical role [1,30,31].

## Conclusion

In summary data presented here show that the highly fusogenic retroviral FRD envelop protein, syncytin 2, is expressed in human placenta throughout pregnancy in some cytotrophoblastic cells, which might be in the fusion stage. Syncytin 2 expression highlights the abnormal trophoblast differentiation observed in placenta of fetal T21-affected pregnancies.

## Methods

### Placenta collection

First trimester placentas were obtained from legal induced abortions (8–12 weeks of gestation). Term placentas were obtained after elective cesarean section from healthy mothers near term with uncomplicated pregnancies. Samples of second trimester placental tissues were collected at the time of termination of pregnancy at 12–25 weeks of gestation (in weeks of amenorrhea) in T21-affected pregnancies (n = 5) and gestational age-matched control cases (n = 5) as previously described [10]. Fetal Down syndrome was diagnosed by karyotyping of amniotic fluid cells, chorionic villi or fetal blood cells. Termination of pregnancy was performed in control cases affected by severe bilateral or low obstructive uropathy or major cardiac abnormalities. The karyotype of placental cells was checked in all cases (free trisomy 21 or normal). The use of these biological samples was approved by our local ethical committee.

### Immunohistochemistry

Placental samples were fixed in 4% formalin for 4–12 h at room temperature and then embedded in paraffin. Briefly, paraffin sections were dewaxed in xylene and rehydrated in ethanol/water. Some sections were classically stained with H&E. Immunostaining was performed with an universal streptavidin-peroxidase immunostaining kit (Dako LSAB, Glostrup, Danemark) using a syncytin 2 monoclonal antibody [18]. All controls, performed with a mouse isotypic IgG1 at the same concentration as the primary antibody were negative.

### Cell culture

For *in vitro *culture villous cytotrophoblastic cells were isolated from second trimester placentas (gestational age-matched controls and T21-affected placentas). 90%–95% of the cells isolated from the normal or T21 placentas were cytokeratin 7-positive. Cells plated in triplicate were cultured for 3 days. Trophoblastic fusion was monitored by desmoplakin immunostaining and nuclei counting as previously described [14].

### Quantification of specific transcripts by real-time RT-PCR

Total RNA was extracted from villous trophoblastic cells cultured for 24 h and 72 h using QIAGEN RNeasy mini kit. cDNA synthesis and PCR amplification were performed as described previously [10]. All PCR reactions were performed using an ABI Prism 7700 Sequence Detection System and the SYBER Green PCR Core Reagents kit (Perkin-Elmer). We used the following primers: FRD (+) 5'-GCCTGC A AATAGTCTTCTTT-3' and FRD (-) 5'-ATAGGGGCTATTCCCATTAG-3'. RNA from the *RPLP0 *gene encoding the human acidic ribosomal phosphoprotein-P0 was used as an internal control and each sample was normalized to *RPLP0 *transcript content. The *RPLP0 *primers used for amplification were: (+) 5'-GGCGACCTGGAA GTCCAAT-3' and (-) 5'-CCATCAGCACCA CAGCCTTC-3'.

### Hormonal assay

The hCG concentration was determined in culture medium at 24 h and 72 h of culture, using an enzyme.

## Competing interests

The author(s) declare that they have no competing interests.

## Authors' contributions

AM performed most of the experimental work and wrote the manuscript. JLF carried out the molecular studies in trophoblastic cells in culture, SB participated in immunostaining, KH participated in immunocytochemistry, PG participated in the isolation of trophoblastic cells, VT participated in placental collection, TH and DEB conceived of the study and participated in its design and coordination and helped to draft the manuscript.

All authors read and approved the final manuscript.
